# Actionable Metabolic Pathways in Heart Failure and Cancer—Lessons From Cancer Cell Metabolism

**DOI:** 10.3389/fcvm.2018.00071

**Published:** 2018-06-19

**Authors:** Anja Karlstaedt, Walter Schiffer, Heinrich Taegtmeyer

**Affiliations:** Division of Cardiology, Department of Internal Medicine, McGovern Medical School, University of Texas Health Science Center at Houston, Houston, TX, United States

**Keywords:** cardiac metabolism, cancer cell metabolism, heart failure, clinical trials as topic, targeted treatments, metabolic targets, systems biology, intermediary metabolism

## Abstract

Recent advances in cancer cell metabolism provide unprecedented opportunities for a new understanding of heart metabolism and may offer new approaches for the treatment of heart failure. Key questions driving the cancer field to understand how tumor cells reprogram metabolism and to benefit tumorigenesis are also applicable to the heart. Recent experimental and conceptual advances in cancer cell metabolism provide the cardiovascular field with the unique opportunity to target metabolism. This review compares cancer cell metabolism and cardiac metabolism with an emphasis on strategies of cellular adaptation, and how to exploit metabolic changes for therapeutic benefit.

## Introduction

The intermediary metabolism of substrates defines every living cell, including heart and cancer cells. Metabolism in mammalian cells has four specific functions: (a) to provide chemical energy from nutrients, (b) to convert exogenous nutrients into macromolecules or building blocks of cell components, (c) to assemble building blocks into proteins, nucleic acid, lipids, and other cell components, and (d) to synthesize or degrade biomolecules required in specialized functions of cells (1). Although intermediary metabolism involves a complex network of pathways, the function of metabolism is remarkably similar in living cells. Recent advances in mass-spectrometry-based proteomics, metabolomics and flux analysis facilitate a more precise dissection of the pathways involved in myocardial dysfunction on a molecular level (2–6). Eventually these tools may lead to actionable and individualized therapies for heart failure patients.

Cardiac metabolism is a dynamic process that adapts to stress by altering its activity to maintain cardiac contraction, thereby ensuring cell survival in the near term. Some metabolic responses shift cardiac metabolism toward an energetically unfavorable state, and turn an initial adaptation into maladaptation, which leads to further disease progression (7–9). Precisely how metabolism affects structural remodeling in the heart, how metabolic activities regulate this transformation, and how metabolic changes can be targeted for therapeutic strategies are key questions under investigation. This review compares cancer cell metabolism and cardiac metabolism with an emphasis on strategies of cellular adaptation, and how to exploit metabolic changes for therapeutic benefit. Here, we have only provided a brief overview on common concepts in the metabolism of heart disease and cancer due to the breadth of literature available in both fields. In cancer cells, the principle applies that alteration in metabolic activities supports the acquisition of biosynthetic material and maintenance of cell proliferation. In other words: reprogrammed metabolism is a hallmark of cancer (10–12). Similarly, alterations in cardiac metabolism contribute to disease progression and severity during cardiac hypertrophy, atrophy, and heart failure (8).

Metabolic adaptation in the heart supports contractile function by maintaining ATP provision. While cancer cells optimize metabolic flux to maximize cell proliferation and growth at pathological levels, the heart tries to optimize the use of energy-providing substrates to ensure cardiac contraction and cell survival. Both cancer cells and the heart show a high metabolic turnover to rapidly adapt themselves to changes in the chemical composition of their exogenous nutrients. The question arises: how do cancer cells successfully improve their cellular fitness by coopting metabolic machinery, while the heart fails to do so under long-term stress? Is it time to seriously rethink cardiac metabolism? To address this question, we will discuss modulation of energy substrate metabolism, biosynthesis of macromolecules, and redox balance in both cancer and the heart below. Regardless of whether specific metabolic activities provide benefits or liabilities to cancer cells or the heart, the rationale is that these activities may be exploited as therapeutic targets. For example, pharmacologic inhibition of fatty acid oxidation by agonists of the peroxisome proliferator-activated receptor (PPAR) ligand-activated nuclear hormone receptors decreases myocardial fatty acid oxidation; which, in turn, promotes increased glucose uptake and oxidation, and improves contractile function (13, 14). There is a strong precedent for using pharmacologic modification of metabolic pathways to improve our understanding and treatment heart diseases and cancer. We postulate that the analysis of metabolic patterns common to human cancers and the failing heart may also provide important insights into the relationships between energy substrates, and lead to metabolic targets in the heart.

## Comparing metabolic pathways in cancer and the heart

We begin our comparison by considering how cancer cells and cardiomyocytes employ pathways that catalyze the degradation of nutrients and the recovery of part of their chemical energy as ATP. The metabolic control of enzyme-catalyzed reactions is tightly regulated in eukaryotic cells through spatial localization, cooperativity, allosteric interactions, substrate availability, expression, and post-translational modification of enzymes. In certain tumors, impairment of mitochondrial function by somatic mutations of Krebs cycle enzymes (e.g., succinate dehydrogenase and fumarate hydratase) leads to activation of glycolysis even in the presence of oxygen (15). Most of these tumors maintain their ability to provide ATP and thrive on glycolysis and glucose oxidation. This phenomenon has been first described by Otto Warburg, who discovered that ascites cancer cells from mice obtain approximately the same amount of ATP from fermentation as from respiration. In fact, limiting the complete oxidation of glucose by inhibiting pyruvate decarboxylation through modulation of pyruvate dehydrogenase activity in tumors fails to prevent tumorigenesis (16). Recent studies in cancer cell lines showed that a switch toward glycolysis is caused by impaired mitochondrial function and regulated by reductive carboxylation of glutamine (17–20). Upregulation of glycolysis allows cancer cells to satisfy their increased demand for biosynthetic intermediates that can be derived from glucose; hence increased glycolysis enables cell proliferation and growth. In the heart, plasma substrate composition and workload dictate nutrient utilization. Under normal physiologic conditions, the heart predominately oxidizes fatty acids (21, 22). However, this substrate preference can quickly shift toward carbohydrates or ketone bodies based on the availability of substrates, the workload, physical activity or periods of starvation. In fact, experimental studies of acutely stressed hearts (21, 23), ischemia and hypertrophy models of transverse aortic constriction (22, 24–26) reveal that glycolysis and glucose oxidation are preferred over fatty acid oxidation. However, this does not mean that the heart is not utilizing carbohydrates under normal physiologic conditions. Both *ex vivo* and *in silico* studies (21, 23, 27) showed that simultaneous oxidation of long-chain fatty acids and glucose allow most efficient ATP provision in the heart during physiologic workload. Degradation of glucose through glycolysis does not only ensure ATP provision, but also provides intermediates for other important pathways, in particular the pentose phosphate pathway and serine synthesis. In tumors and the heart, the glycolytic intermediate glyceraldehyde 3-phosphate is required for the generation of NADPH in the pentose phosphate pathway via glyceraldehyde 3-phosphate dehydrogenase (GAPDH), and anabolic precursors for pentoses and nucleotide synthesis. Flux through the pentose phosphate pathway is regulated by GAPDH, and thus changes in GAPDH activity may impact redox regulation and synthesis of nucleic acids, as well as aromatic amino acids. Recent studies show that tumors exhibiting the Warburg effect are also characterized by increased GAPDH activity (e.g., non-small lung cancers, colorectal cancers) (28, 29, 30, 31). Similarly, during myocardial infarction GAPDH activity increases in the heart and later decreases again during disease progression (32). This change in activity is potentially caused by post-ischemic myocardial reperfusion and may be linked to the production of reactive oxygen species, which have been shown to reduce GAPDH activity in the heart (28, 33). The central role of GAPDH in nucleotide synthesis and generation of reducing equivalents make it critical for survival of cells during stress, and therefore make it a potential target for pharmacologic strategies.

### Fatty acid metabolism

Fatty acids are important metabolic building blocks for membranes to generate acetyl-CoA for post-translational protein modifications [e.g., histone acetylations; (34)], to provide reducing equivalents in the form of NADH and FADH_2_, and to provide ATP through β-oxidation. *De novo* fatty acid synthesis includes several key regulatory enzymes: ATP citrate lyase (ACL) which generates acetyl-CoA from citrate; acetyl-CoA carboxylase which catalyzes the irreversible carboxylation of acetyl-CoA from malonyl-CoA, and fatty acid synthase (FASN) which catalyzes the sequential addition of carbon-units to assemble long-chain fatty acids. In most tissues, including the heart, FASN expression and *de novo* fatty acid synthesis is relatively low, indicating that most cells preferentially take up exogenous or dietary lipids from the blood to provide energy and macromolecule biosynthesis. However, proliferating cells, like several human cancers, have been shown to up-regulate FASN expression (35) and take up free fatty acids to generate phospholipids (36–40). For example, KRAS-driven tumors (e.g., NSCLCs and ovarian cancer) increase fatty acid uptake and oxidation, thereby decreasing the need for *de novo* synthesis. Increased fatty acid oxidation may be driven by increased activation of AMP-activated protein kinase (AMPK) by reduced [ATP]:[AMP] ratio in RAS-mutant cells. Several studies in cancer cell lines and other mammalian tissues showed that ATP and AMP availability regulate the activation of AMPK and the mechanistic target of rapamycin (mTOR), which, in turn, regulate fatty acid metabolism on the molecular level [reviewed by Laplante et al. (41)]. These multiple levels of regulation enable tumors to optimize nutrient utilization and biomass synthesis.

Fatty acid oxidation is a major ATP source for the heart, and depends on cardiac energy demand, oxygen supply and free fatty acid supply from the blood. One of the hallmarks of metabolic perturbations during the development of cardiac hypertrophy and heart failure is decreased use of fatty acids. This metabolic pattern has been observed in both animal and human studies and has been compared to the metabolism in fetal hearts (42–45). Both the fetal and failing heart are characterized by a repression of various genes encoding rate-limiting enzymes of the fatty acid oxidation pathway [e.g., carnithine palmitoyl transferase 1 (CPT1), medium chain acyl-CoA dehydrogenase, and acetyl-CoA carboxylase] (43, 44) and their upstream regulators, including PPARα (46, 47). Downregulation of these genes is not fully understood. However, recent experimental evidence supports the hypothesis that fatty acid oxidation is less efficient (in terms of ATP per oxygen molecule consumed) during mitochondrial dysfunction and limited oxygen availability during ischemic heart disease (48). Therefore, in the short-term this metabolic reprogramming ensures energy provision and cardiac contractile function. In the long-term, reduction of fatty acid oxidation may cause an imbalance between the increased energy demand and simultaneously increased fatty acid availability during heart failure. Several studies have argued that a mismatch between fatty acid uptake and oxidation leads to an accumulation of acetyl-derivatives of CoA and acetyl-CoA as well as carnitine (49), which contributes to cell death and cardiac remodeling (50). In this way, lipotoxicity may contribute to cardiac dysfunction.

### Ketone bodies

In contrast to fatty acid and glucose metabolism, ketone body metabolism has been less investigated in both tumor metabolism and heart failure. Ketone bodies (e.g., acetate, acetoacetate, and beta-hydroxybutyrate) are released by the liver during a wide range of physiologic states; including fasting, starvation, low carbohydrate diets, the neonatal period, post-exercise, pregnancy and diabetes. In extrahepatic tissue (e.g., the heart, brain and skeletal muscle) ketone bodies play an important role for energy provision, post-translational modifications, as signaling mediators, and as modulators of inflammation and oxidative stress. Depending on the tumor type, ketone bodies can either support or diminish cancer cell progression. On the one hand, recent studies showed that ketone bodies support tumor progression and growth in breast cancer and glioblastoma by providing acetyl-CoA for *de novo* lipid synthesis, which is associated with shorter patient survival and increased metastasis of the tumor (51–53). On the other hand, ketogenic diets have been used in animal models and human studies, with potential benefits depending on the tumor location, type and time of diet initiation (54, 55). For example, ketone bodies inhibited growth, proliferation and glycolysis in pancreatic cancer and metastatic glioblastoma cell models and reduced *in vivo* tumor size and attenuated tumor-associated muscle loss (56). Similarly, recent studies indicate that altered cardiac ketone body metabolism contributes to the progression of heart failure (57, 58). These studies provide evidence that ketone body oxidation is increased in the failing heart. However, several questions remain unanswered, including which mechanisms are involved in upregulating ketone body utilization, whether increased use of ketone bodies is adaptive or maladaptive; whether normalization of ketone body oxidation is beneficial or detrimental for the non-failing and failing heart; and whether increased NAD+ levels during ketogenic diets may improve cardiovascular function. Additional work is needed to answer these questions, which will help understanding the role of ketone body metabolism in the pathogenesis of heart failure and evaluating potential risks during cancer treatments.

### Amino acid metabolism

Compared to fatty acids and glucose, amino acids are not predominately used as substrates for energy provision. In general, amino acids are mostly used to provide substrates for protein synthesis or anaplerosis, and function as signaling molecules. For example, aspartate and leucine levels are sensed by mTOR complex 1 at the lysosomal membrane and promote activation of mTOR signaling (59–61). In cancer cells, the contribution of glutamine metabolism to energy provision and tumor progression has been widely studied (17, 20). Reductive carboxylation of glutamine is a common metabolic strategy, which enables cancer cells with somatic mutations of mitochondrial enzymes to maintain growth (e.g., *de novo* lipid synthesis). Furthermore, reductive glutamine carboxylation allows cancer cells to regenerate NADH and NADPH via malate dehydrogenase and/or isocitrate dehydrogenase. In the heart, amino acids predominately serve as fuels for protein synthesis and contribute only in a limited way to ATP provision. Under normal physiologic conditions, amidation of glutamate to glutamine occurs in the heart (62), but glutamine has only a marginal anaplerotic potential and may play a larger role in posttranslational modifications of proteins (e.g., β-linked N-acetylglucosamine) (63). During myocardial anoxia and ischemia, amino acids are used as anaplerotic substrates in the Krebs cycle (64, 65). The question remains whether glutamine plays a similar role in redox regulation in the heart as has been shown in cancer. Metabolomic analysis of failing mouse hearts and human plasma from heart failure patients showed that amino acid levels were increased (66–68). These changes suggest an association between amino acid levels and the progression of heart failure and have been attributed to increased protein breakdown in skeletal muscle. During heart failure, skeletal muscle serves as an additional amino acid source for the heart (69–71). Moreover, several studies suggest that amino acid supplementation helps to increase cardiac function in heart failure. For example, branched chain alpha-keto acids are elevated in hearts of heart failure patients, indicating that breakdown of branched chain amino acids (BCAA) is impaired. However, pharmacologic activation of BCAA catabolism and BCAA supplementation increased BCAA oxidation and improved cardiac contractility in heart failure patients and animal models (67, 72, 73). Cachexia is an independent risk factor for mortality during heart failure (74). With this in mind, improving cardiac amino acid oxidation and protein synthesis in skeletal muscle may protect the heart.

## Biosynthesis and turnover of macromolecules

The biosynthesis of macromolecules is an essential aspect of metabolism in all living cells, as it ensures cellular homeostasis and is essential for cell proliferation and growth. Macromolecules are large molecules, commonly created by polymerization of a smaller subunit. In cells, these smaller subunits are nutrients (e.g., glucose, amino acids, fatty acids), which are converted into biosynthetic precursors through key pathways of intermediary metabolism, including glycolysis, the Krebs cycle, phospholipid pathways, and amino acid synthesis. These biosynthetic precursors then form the three most important biopolymers in the cell: proteins, lipids, and nucleic acids.

Protein biosynthesis is highly regulated in every living cell and requires sufficient supply of essential and non-essential amino acids. A complex network of growth factors, transporters, metabolic intermediates, and cofactors regulate activity of the mTOR signaling system, which is central for the activation of protein synthesis. Somatic mutations of either TSC1 or TSC2 genes causes the formation of hamartomas - a discovery that provided the first molecular link between mTOR and tumorigenesis (75). Phosphorylation and inhibition of TSC2 by AKT promotes activation of mTORC1, which is a common feature of oncogenic deregulation in cancer and may result from PTEN deletion, PIK3CA activating mutations or BCR-ABL translocation (76). Proliferating cancer cells further optimize uptake of amino acids and synthesis of non-essential amino acids through transamination of glutamate. For example, excess glutamine can be exported in exchange for leucine or other essential amino acids, which, in turn, ensures mTORC1 activation (77). At the same time, glutamate uptake and glutaminase activity are stimulated by mTORC1. This bidirectional regulation of mTORC1 activity and the glutamine pool further facilitates protein synthesis in cancer cells.

Protein synthesis and degradation in the heart are highly dynamic processes which are regulated by amino acid availability (78–81), regulation of specific mRNAs, oxygen supply and energy demand. Morgan et al. showed in perfused working hearts that the rate of protein synthesis could be increased by 40% when amino acids levels were increased by five-fold from normal plasma levels (80, 81). These early studies also showed that leucine primarily stimulates protein synthesis. However, overall, the net amount of protein synthesis in the adult heart is low compared to proliferating cells like tumors even when considering substantial increases in protein synthesis during physiologic as well as pathologic hypertrophy of the heart. Cardiac hypertrophy is mediated by protein phosphatases and kinases, such as MAPKs, Janus kinases (JAKs), and the PI3K/PDK/Akt pathway (82, 83). mTOR can be activated by the PI3K/PDK/Akt pathway. Chronic upregulation of mTORC1 is associated with increased cardiac hypertrophy (both physiologic and pathologic) (84, 85). Data from human subjects as well as animal studies show that decreased oxygen in the heart increases glutamine uptake and alanine release into the blood (86, 87). These effects are not seen until oxygen supply is decreased to less than 5% of normal oxygen concentrations, indicating that amino acids are necessary to provide protein precursors during stress (87). Cardiac remodeling is associated with increased glutamine deamination (glutaminolysis) (88). Thus, cardiac glutamine metabolism may enable mTORC1-mediated activation of protein synthesis in a way that mirrors cancer cells.

When nutrients are scarce, two main degradative pathways, autophagy and the ubiquitin proteasome system, enable cells to degrade macromolecules and replenish metabolic intermediates. Autophagy allows cells to maintain homeostasis by delivering protein aggregates and damaged organelles to the lysosome for degradation (89, 90). The formation of autophagosomes is controlled by specific yeast Atg-related proteins, which are tightly regulated by intracellular and extracellular nutrient availability and energy homeostasis of the cell (91–96). Autophagy functions as a cellular stress response that can increase the supply of amino acids by scavenging proteins. However, this contribution is unable to change net protein synthesis or increase nitrogen balance. Therefore, upregulation of glutamine uptake increases the cellular glutamate pool which is required for the synthesis of non-essential amino acids, and thus supports protein synthesis. Tumor cells use extracellular proteins as additional nitrogen source through micropinocytosis. Glutamine metabolism in cancer cells is highly dependent on the tumor type, oncogenic drivers, and the tumor microenvironment (97). Hypoxic tumor regions most distant from nutrient supply upregulate autophagy and sustain mitochondrial glutamine metabolism. Several studies have shown that tumor cells rely on extracellular amino acid supply to sustain cell growth (98). Therefore tumors may employ autophagy, or increase extracellular substrate uptake to buffet growth, while non-cancerous cells rely on autophagy alone during times of stress.

In the heart, autophagy recycles organelles and maintains supply of energy providing substrates during periods of reduced extracellular supply (e.g., starvation), oxygen deprivation (e.g., ischemia), or hemodynamic stress (e.g., valvular heart disease or systemic hypertension) (99–107). Tissue-specific deletion of *Atg5* in heart causes cardiac hypertrophy and contractile dysfunction, indicating that autophagy activation under physiologic conditions is required to maintain cardiomyocytes size and cardiac structure and function (108). Upregulation of autophagy in failing hearts is currently considered an adaptive response to protect cells from stress. However, the role of autophagy in regulating amino acid metabolism in the heart remains unknown, and it remains unclear whether prolonged upregulation of autophagy is beneficial or detrimental in cardiovascular diseases.

*De novo* fatty acid synthesis is required in mammalian cells for membrane biosynthesis, lipidation reactions, signaling pathways and the formation of lipid rafts. Fatty acid synthesis depends on cytosolic acetyl-CoA levels and reducing equivalents in the form of cytosolic NADPH, which is provided through glycolysis via the pentose phosphate pathway. This link between carbohydrate, redox and fatty acid metabolism has been widely studied. Tumor cells in culture use glucose as a source for acetyl-CoA and fatty acid synthesis (19, 109). Most tumor cells also use glutamine, acetate or leucine degradation to enable lipogenesis when glucose availability is reduced, during hypoxia and when mitochondrial function is impaired (17, 34, 110–112). In contrast, the contribution of *de novo* fatty acid synthesis to the lipid pool in the heart is thought to be minimal under physiologic conditions and mostly limited to the synthesis of acetyl-CoA (113–115). The heart is capable taking up complex lipids through lipoprotein particles delivered by the liver (116, 117). Furthermore, glucose is used for glycerol synthesis and storage of fatty acids in the form of triacylglycerides. On the molecular level, Li et al. (118) showed that mTOR complex 1 directly affects *de novo* lipid synthesis through insulin-dependent activation and phosphorylation of S6 kinase (S6K), which then upregulates the sterol regulatory element binding protein 1 (SREBP1) [reviewed by Laplante et al. (41)]. Several questions remain unanswered regarding the metabolic regulation of (i) phospholipid synthesis/turnover, (ii) storage lipids accumulation/utilization, and (iii) cholesterol homeostasis. Recent advances in lipidomics and nutrient flux analysis will help further our understanding of these interconnected processes.

In addition to proteins and lipids, all living cells also rely on the synthesis of nucleic acids (e.g., RNA and DNA) from purines and pyrimidines. Therefore, it is not surprising that nucleotide analogs and antifolates targeting nucleotide biosynthesis have formed an integral part of cancer chemotherapeutic regimens. *De novo* synthesis of purines and pyrimidines requires non-essential amino acids and methyl groups donated from the one-carbon/folate pool. Aspartate and glutamine are required to synthesize the pyrimidine ring. Precursors for nucleotide synthesis are provided by central metabolic pathways including glycolysis, PPP, the serine-glycine pathways, the Krebs cycle and glutamine amidotransferase reactions. The metabolic energy required to enable nucleotide synthesis is substantial and proliferating cells have developed strategies to optimize flux into pathways providing precursors for nucleotides [reviewed by Lane et al. (119)]. The non-essential amino acid, aspartate, is a critical precursor, and most cells generate aspartate through deamination from glutamine rather than through uptake from the blood (120, 121). For many tumors, the rates of aspartate and folate synthesis limit proliferation and growth (122–124). In the heart, aspartate and other amino acids are preferentially used as anaplerotic substrates to provide ATP during ischemia reperfusion injury and cardiac hypertrophy (86, 87, 125). The purine nucleotide cycle in the heart provides fumarate from aspartate to replenish Krebs cycle intermediates (126). Recent reports indicate that purine and pyrimidine metabolism is potentially regulated by signaling pathways, including mTORC1 pathway (127). Depletion of purines, but not pyrimidines, is associated with mTORC1 inhibition, suggesting that in addition to leucine and aspartate, purines may play a role in regulating mTORC1 activity. Additional work is needed to determine how aspartate metabolism, the purine nucleotide cycle and other aspects of *de novo* nucleotide synthesis are regulated in the diseased heart to support protein synthesis as well as energy provision. In all, nucleic acid synthesis represents a rate-limiting step for the growth of tumors, which divert metabolic substrates to maintain cellular proliferation. Conversely, nucleotide metabolism in the heart helps to fuel the Krebs cycle, as there is currently no experimental evidence that its growth depends on nucleotide availability. However, more research may uncover a connection between growth and nucleotide synthesis in the heart.

## Targeting metabolism in the failing heart

Despite striking similarities in the metabolism of the failing heart and cancer cells, there are fundamental differences that need to be considered for the development of pharmacologic treatments. Cancer treatments targeting metabolism have the goal to limit or prevent tumor growth and induce cell death. Interventions targeting cardiac metabolism during heart failure aim to reverse structural remodeling and improve cardiac function. Are there therapies that may target metabolism to reverse heart failure? Are there strategies that both protect the heart and target the cancer? Common pharmacologic strategies for both heart failure and cancer are summarized in Table 1.

**Table 1:** Strategies to target metabolic enzymes for treatment of heart failure and cancer.

**Pathway**	**Effect**	**Compound(s)**	**Target(s)**	**Rationale**	**Cancer field**	**Cardiovascular field**
**GLUCOSE OXIDATION**						
	Inhibition	WZB117	GLUT1	Inhibition of glucose uptake; limiting nutrient supply	Preclinical data only (128, 129)	
	Inhibition	MK-2206	AKT	Inhibition of the PI3K/Akt signaling pathway and cell proliferation; induction of cell apoptosis	Phase II clinical trials (130, 131)	
	Inhibition	Empagliflozin, Canagliflozin	SGLT2	Inhibition of glucose reabsorption by the kidney; limiting nutrient supply	Preclinical data only (132)	In clinical trials (133, 134, 135)
	Inhibition	3-Bromopyruvate, 2-deoxyglucose methyl jasmonate dichloroacetate, clotrimazole and bifonazole, and some traditional Chinese medicinal plants	HK-II	Inhibition of glycolysis to decrease cell growth and survival	Clinical and preclinical data with unacceptable toxicity observed (136)	
	Inhibition	AR-C155858, AZD3965	MCT1, 2 or 4	Inhibition of lactate release, thus promoting increased mitochondrial metabolism; limiting cell growth and survival in cells with upregulated glycolysis and limited mitochondrial metabolism	Clinical and preclinical data (137, 138, 139, 140)	
	Activation		GLUT1			Preclinical data only
	Activation	Dichloroacetate	PDH	used for treating lactic acidosis; in clinical trials for the treatment of pulmonary arterial hypertension, metastatic solid tumors and malignant gliomas	Clinical and preclinical data (141, 142, 143, 144)	Clinical and preclinical data (45, 142, 145)
	Activation	GLP-1	Glucagon analog	Activation of glucose metabolism	Approved 146, 147, 148	
	Activation		HX-II	Activation of glycolysis to increase glucose metabolism		Preclinical data only (149, 150, 151)
**FATTY ACID OXIDATION/ LIPID SYNTHESIS**						
	Inhibition	Trimetazidine, Ranolazine	3-KAT	Activation of glucose metabolism through inhibition of fatty acid metabolism	Approved in Europe and Asia (152, 153)	
	Inhibition	Etomoxir, Oxfenicine, Perhexiline	CPT1-inhibitor	Activation of glucose metabolism through inhibition of fatty acid transport	In clinical trials (Perhexiline); retired due to hepatotoxicity (etomoxir)	Tested in clinical trials; retired due to hepatotoxicity (Etomoxir); limited clinical trials (Oxfenicine, Perhexiline) (154, 155)
	Inhibition	TVB-2640	FASN	FASN is a rate limiting enzyme in *de novo* lipogenesis;	Clinical and preclinical data (137)	Preclinical data only (156, 157)
	Inhibition		GRK2	GRK2 enhances the ERK cascade and promotes partial inactivation of PPARG and FASN inhibition		Preclinical data only (158)
	Inhibition	ETC-1002; BMS303141; SB 204990	ACL	ACL catalyzes the conversion of citrate to acetyl-CoA, and is important for *de novo* lipogenesis	Preclinical data only (159)	
	Activation	ND-630; ND-646; MK-4074	ACC	ACC catalyzes the irreversible carboxylation of acetyl-CoA to malonyl-CoA; ACC inhibition stimulates FAO	Preclinical data only (160)	Preclinical data only (161, 162)
	Activation	Fenofibrate	PPARα	PPARα agonist with antihyperlipidemic activity by activation of lipoprotein lipase and reduction of the production of apoprotein C-III	Approved (163, 164)	
	Activation	Metformin	ETC complex I	Reduction of plasma levels for insulin and IGF-1; Activation of AMPK and inhibition of mTORC1	Approved in T2DM (165, 166)	
**NUCLEIC ACID SYNTHESIS**						
	Inhibition	Methotrexate; pemetrexed	DHFR	Inhibition of DHFR resulting in inhibition of purine nucleotide and thymidylate synthesis; immunosuppressant activities	Approved in various cancer (CVD side effects)	
	Inhibition	5-Fluorouracil	TYMS	Converted to active F-UMP; replacing uracil and inhibits RNA processing	Approved in various cancer (CVD side effects)	
	Inhibition	Hydroxyurea	RNR	RNR required to convert ribonucleoside diphosphate into deoxyribonucleoside diphosphates	Approved in leukemia (CVD side effects)	
	Inhibition	Gemcitabine; Fludarabine	RNR; DNA polymerase	Deoxycytidine analogs are onverted to dFdCDP and dFDCTP which compete with dCTP; prevents nucleotide incorporation	Approved in various cancer (CVD side effects)	
	Inhibition		TKTL1; GAPDH	TKTL1 allows non-oxidative ribose synthesis; GAPDH required for oxidative riobose synthesis and NADPH provision	Preclinical data only	Preclinical data only
**AMINO ACID METABOLISM**						
	Inhibition	Asparaginase	Asparagine availability	Asparaginase hydrolyzes L-aspargine, resulting in inhibition of protein synthesis, cell cyle arrest and apoptosis	Approved in leukemia (CVD side effects)	
	Inhibition	BPTES;CD-839	Glutamine availability	GLS1 inhibition; induces apoptosis, growth arrest and/or autophagy	Preclinical data only	Preclinical data only

*ACC, acetyl-CoA carboxylase; ACL, AKT, protein kinase B; ATP citrate lyase; AMPK, AMP-activated protein kinase; CPT1, carnitine palmitoyltransferase 1; DHFR, dihydrofolate reductase; ERK, extracellular signal-regulated kinase; ETC, Electron transport chain, FASN, fatty acid synthase; GAPDH, glucose-6-phosphate dehydrogenase; GLS, glutaminase 1; GLUT1, glucose transporter 1; GRK2, G protein-coupled receptor kinase 2; HK-II, hexokinase 2; IGF-1, Insulin-like growth factor 1; MCT, monocarboxylate transporter; mTOR, mechanistic target of rapamycin; PDH, pyruvate carboxylase complex; PPAR, peroxisome proliferator-activated receptor; RNR, ribonucleotide reductase; SGLT2, Sodium-glucose co-transporter 2; TKTL1, transketolase-like protein 1; TYMS, thymidylate synthase*.

**Figure 1:**
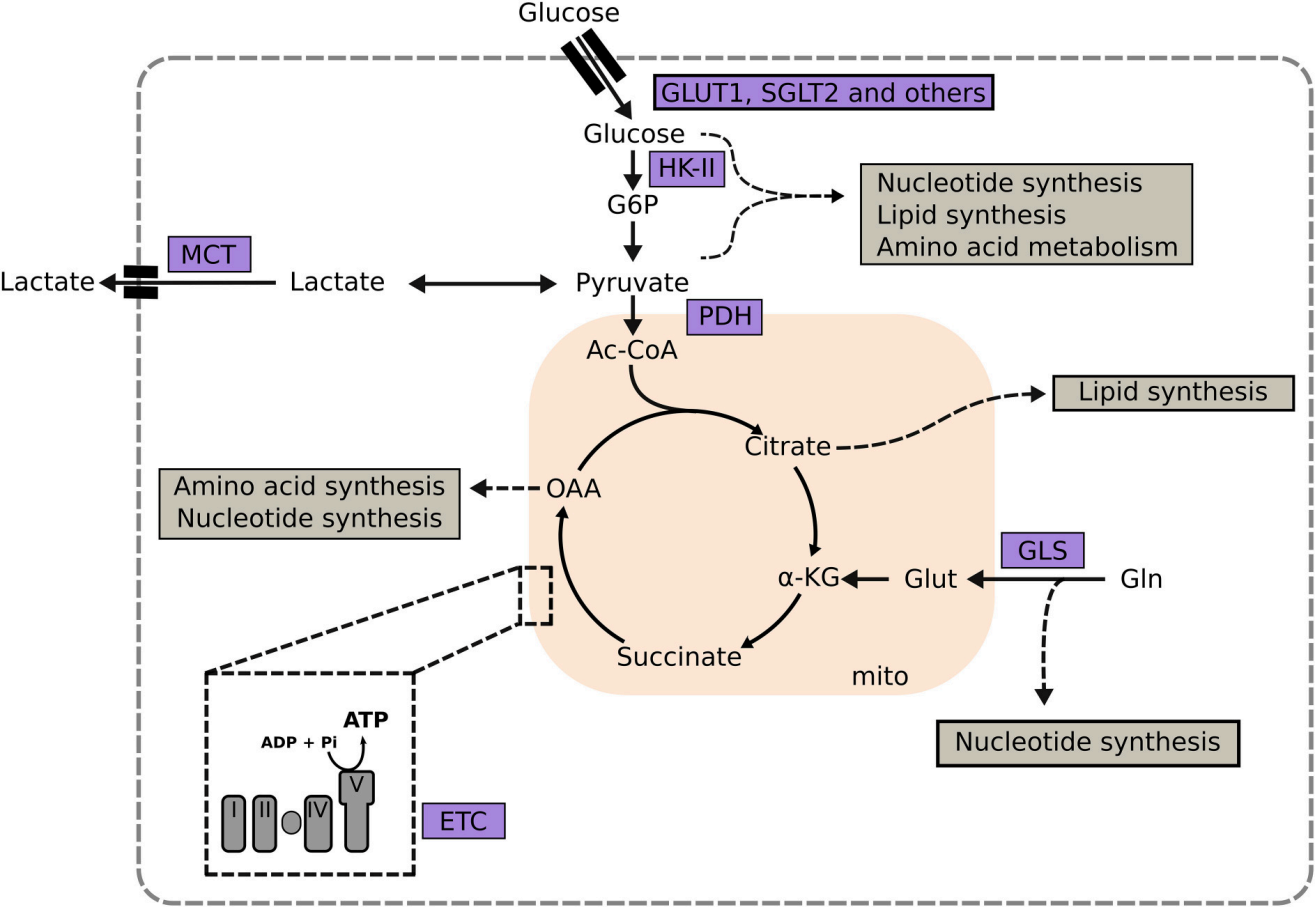
Targeting metabolic enzymes as a strategy in heart failure and cancer. Central metabolic pathways and the involvement of key metabolic enzymes in the synthesis of macromolecules are depicted (shown in gray boxes). α-KG, α-ketoglutarate; G6P, glucose-6-phosphate; GLS, glutaminase; GLUT1, glucose transporter type 1; Glut, glutamate; Gln, glutamine; HK-II, hexokinase II; I, complex I; III, complex III; IV, complex IV; MCT, monocarboxylate transporter; OAA, oxaloacetate; PC, pyruvate dehydrogenase complex; PDK, pyruvate dehydrogenase kinase; Succ, succinate V, complex V.

Glucose metabolism is an attractive target for the treatment of cancer and heart failure, because many solid tumors, as well as the failing heart, upregulate glucose utilization (167, 168). Glucose transporter 1 (GLUT1), a uniporter protein that facilitates glucose transport across the plasma membrane in mammalian cells, is a target for treatment of both cancer and heart failure (Figure 1). Genetic or pharmacologic inhibition of GLUT1 in lung and breast cancer diminishes tumor growth without systemic toxicity (Table 1). In the heart, the opposite approach has been studied. Cardiac-specific overexpression of GLUT1 has been shown to prevent cardiac hypertrophy in a transgenic mouse model subjected to pressure overload by transaortic constriction (169). Intriguingly, inhibition of Sodium glucose co-transporter 2 (SGLT2) in the kidney has proven to be an effective strategy in the treatment of type 2 diabetes with beneficial effects on the heart and at the same time implicated as a potential target in pancreatic and prostate cancers. Thus, depending on the disease progression and tumor type, inhibition of glucose transport may prevent cardiac hypertrophy and reduce cancer growth.

Another potential therapeutic target is Hexokinase II (HK-II; Figure 1 and Table 1), which catalyzes the phosphorylation of glucose to glucose 6-phosphate as the first rate limiting of glycolysis. HK-II binds and inactivates mTORC1 during glucose deprivation, which in turn activates autophagy. Under normal physiologic conditions, mammalian cells predominately express HK-I. Many tumors, including gliomas and NSCLCs, overexpress HK-II making it an attractive metabolic target for pharmacologic inhibitors that disrupt the binding between HK-II and mitochondria. However, HK-II inhibitors showed unacceptable systemic toxicity, e.g., development of cardiac cell death, in clinical and preclinical trials when used at high dosage (170, 171). These observations are supported by heterozygotic HK-II-knockout mouse models, which display increased cardiac susceptibility to ischemia and reperfusion injury, and increased hypertrophy and fibrosis in response to pressure overload (149). In contrast, overexpression of HK II in the heart attenuates cardiac hypertrophy by increasing flux through glycolysis and pentose phosphate pathway (150, 151, 172).

Another potential metabolic target is the PDH complex, which catalyzes the decarboxylation of the glycolytic product pyruvate to acetyl-CoA (Figure 1). The transcription factors c-MYC and HIF induce HK II and pyruvate dehydrogenase kinase (PDK) in a subset of lymphoma, which, in turn, decreases PDH activity. Pharmacologic activation of PDC by the PDK inhibitor dichloroacetate is currently in clinical trials for the treatment of pulmonary hypertension, as well as solid metastatic tumors, and gliomas. The rationale behind this strategy is to promote a tighter coupling between glucose uptake and oxidation. In the heart the premise is to increase ATP provision by increasing complete oxidation of glucose; while in cancer cells that are relying on glycolysis due to mitochondrial dysfunction, dichloroacetate potentially decreases tumor growth, and progression.

Fatty acid and mitochondrial metabolism have also emerged as targets for treatment of heart failure and cancer (173). The rationale in cancer treatment is to limit tumor proliferation by inhibiting *de novo* lipogenesis or stimulating fatty acid oxidation. Intriguingly, pharmacologic strategies when targeting fatty acid metabolism have been similar in heart disease and cancer. Modulation of fatty oxidation by selective inhibition of 3-ketoacyl coenzyme-A thiolase (3-KAT) and CPT1 have been either approved (e.g., 3-KAT inhibitors like trimetazidin) or tested in clinical trials for both heart failure and cancer (Table 1). However, application of CPT-1 inhibitors is limited due to hepatotoxicity and other severe side effects. Other approaches focus on limiting *de novo* lipid synthesis by inhibiting FASN or ATP citrate lyase (ACL). FASN is the rate limiting enzyme for *de novo* lipogenesis, while ACL catalyzes the conversion of glucose-derived citrate to acetyl-CoA and regulates cytosolic acetyl-CoA levels. Similarly, pharmacologic inhibition of GRK2 partially inactivates PPARγ and inhibits FASN through mitogen-activated protein kinases (MAPK) (158). Ongoing clinical trials indicate the efficacy for FASN inhibition in cancer. Similar trials in heart failure have not been successful. Another common metabolic target that may be employed is the electron transport chain (ETC), and specifically metformin, which has been increasingly used as an anti-cancer agent (174–177). By inhibiting ETC complex I (Figure 1), metformin decreases mitochondrial ATP provision (120, 178). In cancer cells, this inhibition increases the reliance on glycolysis for ATP provision, and makes cancer cells vulnerable when glucose availability is limited. Additionally, metformin reduces plasma levels of insulin and insulin-like growth factor 1 (IGF-1), which further constricts glucose availability to glycolysis-dependent cancer cells.

A further potential treatment strategy is targeting nucleic acid synthesis and amino acid metabolism. Among the various pharmacologic agents targeting nucleic acid synthesis that are available for cancer therapy, almost all have been reported to have cardiovascular side effects. Glutaminase inhibitors offer a potential way to inhibit mitochondrial amino acid metabolism (Figure 1), to induce apoptosis, growth arrest and autophagy. Certain tumors (e.g., NSCLCs and pancreatic tumors) show increased uptake and utilization of glutamine to support macromolecule synthesis and ATP provision (18, 179–181). Prolonged activation of autophagy may be involved with disease progression and decreased cardiac contractility (182, 183); thus, glutaminase inhibitors can reduce tumor burden and potentially improve cardiac function during advanced stages of heart failure.

## Outlook and conclusions

We presented several common metabolic strategies that both cancer cells and cardiomyocytes employ to optimize nutrient flux and cell growth. Metabolic reprogramming is a hallmark of both heart failure and malignant cells, which provides them with the ability to survive and sustain stress. Recent progress in molecular techniques (e.g., CRISPR/Cas9) and metabolic flux analysis using stable isotope labeling improved our understanding of mechanisms, biological consequences, and vulnerabilities associated with metabolic reprogramming in heart disease and cancer. Somatic mutations in metabolic reprogramming predominately stems from redirections of metabolic intermediates and increased ATP demand in the context of decreased cardiac contractility. Intermediary metabolites serve as signals that activate signaling pathways, modulate posttranslational modifications of proteins and alter gene expression. Examination of these relationships has inspired pharmacologic strategies that aim to either correct or enhance metabolic vulnerabilities in cancers and the failing heart. In cancer, potential pharmacologic targets manifest in pathways that regulate energy homeostasis and macromolecule biosynthesis. In the heart, similar strategies are often accompanied by severe side effects and increased cell death. Developing rational therapeutic strategies for both cancer and cardiovascular diseases will be aided by integrating findings on a systems level from pre-clinical and clinical studies. Little is known about the metabolic interaction between tumors and the heart. However, recent studies show that oncometabolic dysregulation can promote cardiac dysfunction (27). Despite the vast metabolic differences and functions of cancer cells and the heart, their common metabolic requirements present opportunities to find intersections for new therapies. Recent experimental and conceptual advances in cancer cell metabolism [reviewed by Vander Heiden et al. (184)] provide the cardiovascular field with the unique opportunity to target metabolism. This strategy holds the potential for new therapies to combat heart failure, as well as chemotherapies that may protect the heart as much as they subvert cancer.

## Author contributions

AK drafted and wrote the manuscript. WS edited the manuscript, critically evaluated and searched the literature. HT provided guidance and edited the manuscript.

### Conflict of interest statement

The authors declare that the research was conducted in the absence of any commercial or financial relationships that could be construed as a potential conflict of interest. The reviewer XP and handling Editor declared their shared affiliation.
